# Mining online e-liquid reviews for opinion polarities about e-liquid features

**DOI:** 10.1186/s12889-017-4533-z

**Published:** 2017-07-07

**Authors:** Zhipeng Chen, Daniel D. Zeng

**Affiliations:** 1 Department of Management Information Systems, University of Arizona, 1130 East Helen Street, Tucson, 85721-0108 USA; 2 The State Key Laboratory of Management and Control for Complex Systems, Institute of Automation, Chinese Academy of Sciences, Beijing, People’s Republic of China

**Keywords:** E-cigarette, E-juice, E-liquid, Flavors, VG, PG, Nicotine, Cloud production, Throat hit, Online product review

## Abstract

**Background:**

In recent years, the emerging electronic cigarette (e-cigarette) marketplace has developed prosperously all over the world. By analyzing online e-liquid reviews, we seek to identify the features attracting users.

**Methods:**

We collected e-liquid reviews from one of the largest online e-liquid review websites and extracted the e-liquid features by keywords. Then we used sentiment analysis to classify the features into two polarities: positive and negative. The positive sentiment ratio of a feature reflects the e-cigarette users’ preference on this feature.

**Results:**

The popularity and preference of e-liquid features are not correlated. Nuts and cream are the favorite flavor categories, while fruit and cream are the most popular categories. The top mixed flavors are preferable to single flavors. Fruit and cream categories are most frequently mixed with other flavors. E-cigarette users are satisfied with cloud production, but not satisfied with the ingredients and throat hit.

**Conclusions:**

We identified the flavors that e-cigarette users were satisfied with, and we found the users liked e-cigarette cloud production. Therefore, flavors and cloud production are potential factors attracting new users.

## Background

E-cigarette usage has been increasing rapidly [1, 2], but it is still a controversial product. Previous research has reached mixed conclusions regarding the benefits or risks of e-cigarettes. On one hand, e-cigarette products usually contain less harmful chemicals than regular cigarettes and are regarded as an efficient method for tobacco consumption reduction and abstinence [3–5]. On the other hand, young people could get addicted to the nicotine in many e-cigarettes, which could result in the adoption of other tobacco products like regular cigarettes [6].

In this paper, we seek to identify what e-cigarette features attract consumers based on the feature-level sentiment analysis of online e-liquid reviews. E-liquid is the liquid mix used in e-cigarette kits to produce the smoke and cloud. JuiceDB is one of the world’s largest review websites of e-liquid. Different from the posts from other mainstream social media like Twitter and Reddit, JuiceDB posts are more focused on the e-liquid features [7]. The rich information in the review data and advanced sentiment analytics have made mining and summarizing e-cigarette information from the unstructured texts possible [8].

Prior studies identified flavor as a factor that is attractive to e-cigarette adopters [9–11]. Fruit flavors are the most popular among users, while tobacco flavors are preferred for initial e-cigarette use among current smokers [12, 13]. Tobacco companies have already successfully applied flavor varieties to the marketing of traditional tobacco products to young people, which resulted in banning cigarette flavors except for menthol [14]. Therefore, e-cigarette flavors have become one of the FDA’s concerns because of the influence on youth adoption and disease susceptibility [15]. However, currently we only have information on the popularity of flavors, not the users’ attitudes towards different flavors in a fine-grained flavor analysis. There is little research about other e-liquid features [16] and users’ attitudes towards them. Despite the growing amount of literature on e-cigarettes on YouTube [17, 18], Twitter [19], Reddit [12], online forums [20] and multiple platforms [7], there are no published studies that have systematically summarized and analyzed the popularity and preference of e-liquid features based on online review websites. We distinguished preference from popularity in the analysis of e-liquid: popularity is how often the e-cigarette users discuss about specific e-liquid features, but preference is whether the users like the e-liquid features and measured by positive sentiment ratio in our study. This paper reveals the favorite flavor lists and the attitudes towards e-liquid ingredients, cloud production and throat hit based on the analysis of e-liquid reviews. The results indicate flavors and cloud production are the attractive features of e-liquid. Thus, it provides complementary insights about e-liquids and has implications for healthy behavior promotion and e-cigarette regulations. The data-driven findings could benefit regulatory agencies and e-cigarette control organizations so that they can develop better campaign or policies to suppress the e-cigarette attractiveness to non-smokers and boost the effect of e-cigarettes on tobacco control.

## Methods

### Data

E-liquid reviews were collected from JuiceDB from June 26, 2013 to November 12, 2015 for study purposes. JuiceDB is one of the world’s largest independent review websites of e-liquids and vape juices. It claims to have more than 17,000 reviews and 14,000 registered users. Each review includes the author’s account, e-liquid name, brand, ratings, and detailed comments. The ratings are integers ranging from one to nine at the time we collected data. In total, we collected 14,433 e-liquid reviews.

### Data analysis

To gain a systematic understanding of which e-liquid features the e-cigarette users care about and their feelings about these features, we first extracted e-liquid feature texts from e-juice reviews by keyword search, then conducted sentiment analysis on the feature texts to reveal the opinion polarities.

#### Feature text extraction

First, we identified three aspects of e-liquid features: flavors [7, 12, 13], common ingredients [7, 21] and smoking feelings [7]. Previous studies listed and categorized e-liquid flavors [12, 13]. We followed the flavor categorization and manually identified more flavors mentioned in the reviews, including pear, plum, grape and lime in fruit category, cheese and butter in cream category, and caramel in sweet category. The basic e-liquid ingredients are water, nicotine, flavorings, vegetable glycerin (VG) and propylene glycol (PG). Nicotine, VG and PG are frequently discussed in posts. Nicotine is widely contained in tobacco products. Its users are affected physically and easily get addicted. VG increases the flavor and creates large amounts of vapor, and PG produces a great throat hit. The typical ratio of PG and VG is 50/50, 60/40 and 70/30. We manually identified two smoking feelings: cloud production and throat hit. These two features are discussed online [21] and also mined by topic analysis [7]. Cloud production means how much cloud the e-liquid can produce, and the throat hit is the feeling at the throat when using e-cigarettes. These two features are manipulated to imitate the traditional cigarette or cigar experience, and many e-cigarette users enjoy the cloud and the throat hit. All the features are listed in Table 1.

**Table 1:** Sentiment analysis of single flavors, ingredients and smoking feelings

Category		Frequency	Positive sentiment ratio	Feature	Frequency	Positive sentiment ratio
Flavor	Fruit	7811	0.845	Strawberry	1634	0.862
				Banana	992	0.871
				Apple	1177	0.838
				Blueberry	684	0.855
				Mango	243	0.811
				Cherry	273	0.747
				Orange	420	0.714
				Lemon	629	0.797
				Watermelon	429	0.821
				Raspberry	363	0.835
				Pomegranate	143	0.909
				Pear	666	0.890
				Plum	110	0.873
				Grape	267	0.801
				Lime	439	0.897
	Cream	6372	0.872	Cream	3978	0.886
				Vanilla	1506	0.893
				Custard	921	0.893
				Milk	886	0.871
				Chocolate	691	0.855
				Cake	651	0.848
				Cookie	299	0.823
				Cheese	151	0.828
				Butter	862	0.869
	Tobacco	1291	0.802	Tobacco	1291	0.802
	Menthol	1369	0.845	Menthol	891	0.820
				Mint	806	0.880
	Beverages	1244	0.830	Coffee	521	0.856
				Tea	654	0.806
				Wine	49	0.857
	Sweet	2642	0.854	Candy	1357	0.832
				Honey	657	0.878
				Caramel	748	0.873
	Seasonings	1145	0.853	Cinnamon	958	0.871
				Pepper	204	0.750
	Nuts	1697	0.878	Nuts	1698	0.878
Ingredients				Nicotine	595	0.780
				PG	728	0.713
				VG	1365	0.737
Smoking feelings				Cloud production	993	0.871
				Throat hit	1677	0.770

Second, we used feature keywords to extract sentences about the features of interest. Shown in Table 1, the features in flavor category are specific flavors and they are categorized into eight subcategories. The keywords of cream, tobacco, menthol, sweet and nuts subcategories only include the corresponding features because they are also specific flavors. Fruit, beverages and seasonings are not specific flavors but categories only, so when calculating the popularity and preference of these subcategories, the keywords do not only include the corresponding flavors but also include themselves. For example, the keywords of beverages subcategory are beverages, coffee, tea and wine. The keywords of ingredients and smoking feelings are also the corresponding features. After extracting sentences by keyword searching, the feature sentences from each review form the feature texts for sentiment analysis.

#### Sentiment analysis

We applied sentiment analysis to classify the feature texts into two categories: positive and negative. If a text is in the positive sentiment category, the review text writer likes the feature; if a text is in the negative sentiment category, the text reflects the review writer doesn’t like the feature. Because the dataset is product reviews, all posts are very emotional; therefore, we don’t have the neutral category. Many posts have mixed sentiment, but the users usually have overall evaluations on the e-liquid. Thus, we don’t have a category for mixed sentiment but consider whether the overall sentiment is positive or negative.

We manually labeled 500 randomly selected posts. The sentiment label is consistent with review ratings (correlation = 0.72). If we regard the reviews with ratings higher than 7 as positive and the reviews with ratings equal to or lower than 7 as negative, the agreement and Krippendorff’s alpha are maximized (agreement = 91.2%, Krippendorff’s alpha = 0.71). As the review ratings objectively reflect the users’ likes or dislikes, we chose them as ground truth and regarded the reviews with ratings higher than 7 as positive and the reviews with ratings equal to or lower than 7 as negative. About two-thirds of the reviews were deemed to be positive.

Then we trained a NBSVM sentiment analysis model, which integrates Naive Bayes and Support Vector Machine and achieves good performance on texts of different lengths [22], on the training dataset including 3000 randomly selected reviews. In the training set, 2097 reviews are positive and 903 reviews are negative. We used the remaining 11,712 reviews as the test dataset and achieved an accuracy of 82.04%. To further test the effectiveness when applying this classifier to short texts, we manually labeled 150 sentences from the reviews, and the testing accuracy is 72.67%. Therefore, this classifier is reliable for sentiment analysis no matter whether the feature texts are long posts containing multiple sentences or just single sentences.

## Results

### Single flavors

We counted the number of times that each flavor category and flavor word occurred in the reviews. If a review mentioned several flavors, all the flavors mentioned are counted once. Table 1 shows the breakdown of reviews for each flavor category and specific flavor. The flavor frequency is the total number of reviews mentioning the flavor, and the category frequency is the total number of reviews mentioning any flavor in this category. The frequency indicates the flavor or category *popularity*. higher frequency of a flavor means it is more *popular*. The positive sentiment ratio indicates the ratio of reviews with a positive sentiment about the flavor, which means the user *preference*. Higher positive sentiment ratio means a flavor is more *favored* by users. The total number of flavor frequencies is 31,717. Thus, the average of flavors per review is 2.20.

The popularity and preference are moderately correlated (correlation = 0.31), so the popularity of certain flavors doesn’t imply the e-cigarette users like them. Fruit and cream are the most popular flavor categories, while seasonings, tobacco and beverages are the least mentioned. Cream and nuts have the highest positive sentiment ratios, i.e., they are the most favored, while tobacco and beverages are the least favored with the lowest positive sentiment ratios. Though fruit is the most popular flavor category, some fruit flavors are not widely favored, such as cherry, orange, and lemon, as their positive sentiment ratios are low. Among all flavors mentioned more than 500 times, the favorite flavor list includes vanilla, custard, pear, cream and mint.

### Ingredients and smoking feelings

The frequency and preference of nicotine, PG and VG are shown in Table 1. The users discuss less about nicotine than VG and PG, though nicotine is known for its addictive power. Besides, they are less pleased with these three ingredients than most flavors. The analysis results also show the users are satisfied with cloud production, so cloud production is one reason the users like e-cigarettes. However, the users are not satisfied with throat hit. This suggests the cigarette throat hit is better than e-cigarette throat hit for the users.

### Mixed flavors

**Fig. 1:**
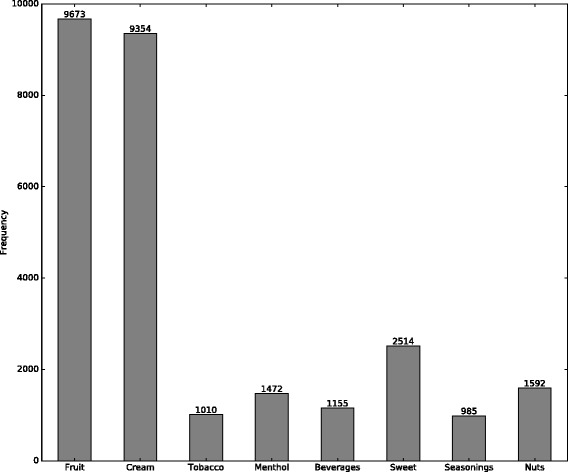
Category frequencies in mixed-flavor reviews

Many e-liquids combine two or more flavors. The 7,736 reviews that mentioned multiple flavors were analyzed to examine popular patterns of mixed flavors. The flavor frequency is the number of reviews mentioning the flavor, and the category frequency is the sum of the flavor frequency in this category. Figure 1 presents how many times each category is mentioned in mixed-flavor reviews. It shows that flavors in fruit and cream categories are most often used in mixed flavors.

**Fig. 2:**
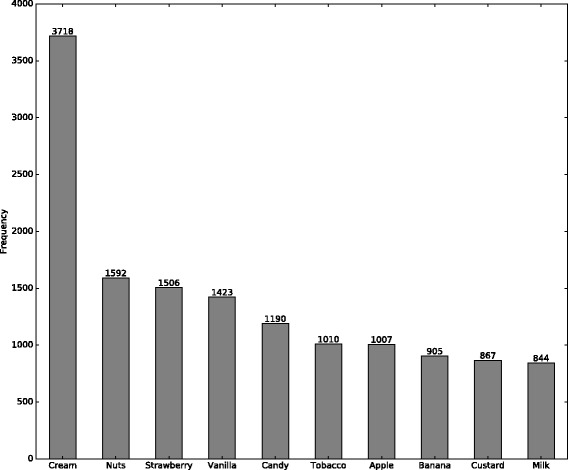
Most popular flavors in mixed-flavor reviews

We also examined specific flavors in mixed flavors. As shown in Fig. 2, cream is the most popular flavor, followed by nuts, strawberry and vanilla. The top 10 popular flavors in mixed-flavor reviews include three fruit flavors and four cream flavors, which is consistent with the findings about category popularity in mixed-flavor reviews.

The most popular combinations of two flavors and three flavors were also analyzed. The popularity and preference of mixed flavors are barely correlated. The correlation for two-flavor combinations is 0.08 and the correlation for three-flavor combinations is 0.02. The top co-occurrences of flavors are listed in Table 2. The most popular combinations are quite different from the most favored combinations, although both the most popular and the most favored combinations contain strawberry, cream, vanilla, custard, nuts and milk. Comparing Tables 1 and 2, we found that the top mixed flavors are preferable to the single flavors.

**Table 2:** Most popular or favored mixed flavors

Mixed flavor	Frequency	Positive sentiment ratio
Most popular combinations of two flavors		
Strawberry + cream	745	0.881
Cream + vanilla	701	0.919
Cream + nuts	645	0.921
Cream + milk	544	0.881
Cream + custard	514	0.916
Banana + cream	482	0.905
Cream + butter	403	0.883
Vanilla + custard	397	0.899
Cream + candy	369	0.881
Cream + cinnamon	353	0.907
Most popular combinations of three flavors		
Cream + vanilla + custard	219	0.927
Strawberry + cream + milk	203	0.877
Cream + butter + nuts	162	0.907
Vanilla + tobacco + caramel	137	0.854
Banana + cream + nuts	130	0.946
Most favored combinations of two flavors (frequency >100)		
Milk + nuts	118	0.958
Pear + honey	161	0.950
Banana + butter	128	0.945
Custard + milk	107	0.944
Strawberry + nuts	123	0.943
Lime + cream	105	0.943
Cream + mint	108	0.935
Vanilla + nuts	225	0.933
Vanilla + chocolate	102	0.931
Vanilla + milk	101	0.931
Most favored combinations of three flavors (frequency >90)		
Banana + butter + nuts	101	0.950
Cream + vanilla + caramel	116	0.948
Banana + cream + nuts	130	0.946
Cream + vanilla + nuts	96	0.938
Cream + vanilla + custard	219	0.927

## Discussion

To the best of our knowledge, this is the first systematic analysis on e-cigarette features by mining online reviews. In this research, the popularity of an e-liquid feature was defined as the number of posts mentioning the feature and the preference was measured by positive sentiment ratio of polarity sentiment analysis. We summarized popularity and preference of flavors, ingredients and smoking feelings.

The flavor popularity results are consistent with previous literature [12]. Our study shows the favorite flavor list is different from the popular flavor list. The most popular flavor category is fruit, followed by cream, but the most favorite flavor category is nuts, followed by cream. Though fruit-flavored e-liquid is widely produced and sold, some fruit flavors, such as cherry, orange, and lemon, are not favored by many users. In contrast, the flavor of nuts is one of the popular single flavors, and is also popular in mixed flavors. The top favorite flavors mentioned more than 500 times include vanilla, custard, pear, cream and mint. The unfavorable tobacco flavor implies the flavor variety of e-liquid is one of the key factors of the e-cigarette sales growth and possibly attracts non-smokers. Besides, the top mixed flavors are preferable to single flavors. Fruit and cream categories are most often mixed with other flavors. The flavors occurring most often are cream, nuts, strawberry and vanilla.

We also analyzed other e-liquid features: cloud production, throat hit, nicotine, VG and PG. To the best of our knowledge, this is the first analysis of users’ opinions about these features based on online review data. The users are satisfied with cloud production, so this feature is likely to be another reason of e-cigarette consumption. On the contrary, the users feel less satisfied with the ingredients and throat hit. Besides, the users are less concerned about nicotine than flavors, smoking feelings, VG and PG, though nicotine is highly related to addiction and health issues. Much fewer discussions on nicotine than flavors and smoking feelings may suggest a hedonic consumption tendency of e-cigarettes. Previous literature suggests the ingredients are related to some symptoms, such as balanced to high VG level related to cough and high PG related to throat harshness [21]. These are consistent with our results that the users are not satisfied with VG and PG. Therefore, the preference of e-cigarette users can reflect the health issues of the ingredients and act as an indicator of e-cigarette safety. by tracking the user preference, the policymakers can discover potential risks of e-cigarettes, and the e-cigarette producers can also improve the e-cigarette safety.

In summary, both flavor variety and cloud production are important factors attracting e-cigarette users. This analysis of JuiceDB data is an important step in understanding the consumption choice of different e-cigarette products and could lead to continuous observations of emerging e-liquid trends. Consumers, e-cigarette producers, policy makers and health organizations could make use of this information to improve e-cigarette products and leverage e-cigarettes to control tobacco.

Our study has limitations. First, our study is based on JuiceDB posts. This dataset is not likely to cover all opinions on e-liquid and it probably includes advertisement posts. However, JuiceDB as an online review website has the richest data narrowly on user experience and evaluation on e-liquid features [7], and the popularity results are consistent with the analysis on Reddit data [12]. Besides, the dataset 14,433 reviews is large enough, so we believe JuiceDB dataset is representative and the analysis results are reliable. Second, it is possible that a broader range of data would provide a more comprehensive understanding of users’ opinions about e-cigarette products, but we believe the dataset in this study can support our conclusions. Third, user profiles are not available in JuiceDB; hence we could not identify the preference patterns of specific user groups.

Further studies are still needed to reveal users’ opinions on e-cigarettes and the influence of e-cigarettes on users. We envision further opinion extraction from online reviews to improve our understanding of users’ experience and attitudes. Besides, the ingredients interact with each other to produce health effect. The safety of composition of e-juice ingredients should be further explored. Our study only considered the common ingredients from the users’ subjective opinions. The symptoms or adverse effects of the e-cigarette ingredients including all kinds of flavorings should be examined by clinical study to gain solid ground truth of the effect of using e-cigarette.

## Conclusions

This study shows that review websites are heavily used by the e-cigarette and vaping community to share information about every aspect of e-liquid use and that the e-liquid review data can be mined for valuable information on self-reported attitudes and opinions. We proposed to use preference instead of popularity to reveal e-cigarette users’ attitudes to e-liquid features. Nuts and cream categories are favorite flavors, and they are also popular and welcomed in mixed flavors, along with strawberry from the fruit category. Furthermore, we found the users are satisfied with the cloud production of e-liquids, but not the e-liquid ingredients or throat hit. Thus, flavors and cloud production are potential factors attracting new users.

## Abbreviations

FDA: the U.S. Food and Drug Administration
PG: propylene glycol
VG: vegetable glycerine
